# Comparison of traffic-injury related hospitalisation between bicyclists and motorcyclists in Taiwan

**DOI:** 10.1371/journal.pone.0191221

**Published:** 2018-01-17

**Authors:** Chih-Wei Pai, Hsiao-Yu Lin, Shin-Han Tsai, Ping-Ling Chen

**Affiliations:** 1 Graduate Institute of Injury Prevention and Control, College of Public Health, Taipei Medical University, Taipei, Taiwan; 2 Department of Urology, Taipei Medical University Hospital, Taipei, Taiwan; 3 Department of Emergency Medicine, Shuang Ho Hospital, New Taipei City, Taiwan; University of New South Wales, AUSTRALIA

## Abstract

**Objectives:**

Bicyclists and motorcyclists contribute substantially to the morbidity and mortality rates of road crash casualties. The objective of the study was to investigate the crash characteristics of bicyclist and motorcyclist casualties presented to hospitals in Taiwan resulting from crashes.

**Methods:**

By using linked data from The National Traffic Crash Dataset and the National Health Insurance Database between 2003 and 2012, logistic regression models were used to examine the determinants of hospitalisation among motorcyclist and bicyclist casualties. The examined variables include demographic characteristics, road and weather conditions, and vehicle characteristics.

**Results:**

A total of 1,998,606 two-wheelers were enrolled in the study, of whom 216,600 were hospitalised: 203,623 were motorcyclists and 12,964 were bicyclists. Bicyclists were more likely to be hospitalised than motorcyclists were (14.0% vs. 10.7%). The pooled logistic regression model shows that bicyclists had higher odds of hospitalisation than motorcyclists (adjusted odds ratio [AOR] = 1.11, 95% confident interval [CI] = 1.08–1.14). In the motorcyclist and bicyclist models, helmet non-use appears to be a determinant of hospitalisation for motorcyclists (AOR = 1.14, CI = 1.12–1.16), although insignificant for cyclists (AOR = 1.03, CI = 0.94–1.12). Other important determinants of hospitalisation for motorcyclists and cyclists include female riders, elderly riders, rural roadways, unlicensed riding (for motorcyclists only), curved roadways, defective roadways, alcohol consumption (only for motorcyclists), and single-vehicle crashes (for motorcyclists only).

**Conclusions:**

The result that bicyclists had an increased probability of being hospitalised than motorcyclists is particularly noteworthy, because there have recently been much more users of bikesharing systems in metropolitan cities where cycle helmets are not provided. We further found that helmet non-use was also a risk factor for motorcyclists, but insignificant for cyclists, possibly due to lower helmet utilization rates among bicyclists. Our findings regarding the increased hospitalisation percentage emphasize the importance of helmet use.

## Introduction

The size of Taiwan is 36,197 square kilometres, with a population of 23,552,470. There are a total of 7,870,102 registered cars and 13,690,684 registered motorcycles, with the equivalent of 217 cars/km^2^ and 378 motorcycles/km^2^. Road traffic-related crashes are the leading cause of injuries requiring hospitalisation [1]; bicyclists and motorcyclists substantially contribute to the morbidity and mortality rates of road crash casualties [2]. Traffic crashes involving two-wheeled vehicles (including motorcycles and bicycles) have been recognized as a serious problem in Taiwan because of their high severity. Motorcyclist fatalities in Taiwan contribute to the most traffic deaths [3]. In 2013, official statistics showed that motorcyclists account for 51% of the total traffic fatalities, and the numbers of fatal injuries were 20 times higher among motorcyclists than among automobile drivers [4]. On average, one motorcyclist death occurs every 24 h in Taiwan [4]. As many as 6.74% of traffic fatalities are bicyclists (which increased from 3.55% in years 2005) [4].

Past studies have suggested that head injuries are the primary cause of deaths and hospitalisation among motorcyclists and bicyclists [5–7]. Studies conducted on motorcyclist injuries have reported that helmet use and related laws have successfully reduced head injuries, thus reducing fatalities among motorcyclists. Ichiwaka et al. [8] reported a 41% reduction in head injuries in Thailand 2 years after the implementation of a mandatory helmet use law. A similar reduction in head injuries and fatalities has been reported in Malaysia [9], Vietnam [10], the United States [7, 11], and Italy [12] after the implementation of helmet use laws. Past studies of the effect of motorcycle and bicycle helmet on injured body regions were systematically reviewed [13, 14]. It was concluded that helmet use was associated with reduced odds of death and head injury. The meta-analysis by Olivier and Creighton [13] reported that bicycle helmet use was associated with a reduced risk of head injury by 51%, serious head injury by 69%, face injury by 33%, and fatal head injury by 65%. Another meta-analysis study National helmet laws for motorcyclists became mandatory in 1997 motorcycle helmet concluded that motorcycle helmet use was estimated to reduce the risk of death by 42% and head injury by 69% [14]. The effect of helmet use on neck injury was rather inconclusive. Bicycle helmet use is a means of reducing morbidity and mortality among bike users. Several case-controlled studies have reported an association of helmet use with a decreased rate of head injury and mortality among riders of all ages, with bicycle helmets reducing the risk of head and brain injury by 65%-88% [15]. Moreover, Attewell et al. [16] conducted a meta-analysis of 16 observational studies and reported that bicycle helmets can significantly reduce the risks of head injury by approximately 60%.

Aside from examining the effects of helmets on head injuries and fatalities, past studies have attempted to investigate other influential factors on motorcyclist and cyclist injury severity. Several important findings have been concluded in past studies of motorcyclist injury severity: injuries sustained by motorcyclists tend to be more severe with the increasing engine size of motorcycle [17], when the rider was a female [18, 19], an elderly [20], when the rider had recessive speed and was drink-riding [20, 21], when the crash occurred in rural area and on weekends [21], when struck by a heavier vehicle [22], and when the rider was involved in an approach-turn/head-on crash [21].

Important determinants of bicyclist injury severity reported in the literature include: a head on/angle crash [23–26], speeding-involved [23], truck involved [23–25, 27, 28], an elderly cyclist [23, 27], driver/cyclist intoxication [23, 25, 28], vertical/horizontal curves [25, 26, 28], night without streetlight [23, 24, 26], higher speed limit [24, 26, 27].

The Taiwan police-reported crash dataset revealed a steady decrease in the number of motorcycle crashes but a stable increase in that of bicycle crashes, possibly because of the increasing popularity of bicycle use. For instance, bikesharing systems have been implemented in several metropolitan cities in Taiwan, such as Taipei City, Taoyuan City, Taichuang City, and Tainan City. The fatality rate among bicyclists is two times that of motorcyclists, mainly because of head injuries, which account for approximately 50% of all bicyclist fatalities [4]. National helmet laws for motorcyclists became mandatory in 1997, and there is no province level variation in helmet laws. By contrast there are no mandatory helmet laws for bicyclists. As a result helmet utilization rates are 90.6% and 8.5% among motorcyclists and bicyclists, respectively [29].

Based on our review of previous research, a common criticism of most previous studies is that they tended to rely on employing either police reported data (e.g., [26]) or hospital data alone (e.g., [30]). As a result, significant gaps remain in understanding the thorough relationship between various demographic, environmental, and crash characteristics and the entire injury-severity spectrum. These studies relying on police-reported data suffer from a data constraint: injury information reported by police tends to be less reliable [31]. For those using hospital data alone, their results may not represent the whole population and were limited to a certain data-collection period (see, for instance, the study by Cunningham et al., 2012) [32]. In addition, classification of road user, i.e., either a motorcyclist or a cyclist, in hospital datasets tends to be incomplete. This study fills an important gap in the literature by investigating the thorough relationship between various factors and motorcyclist/cyclist injury severity using linked data as opposed to relying on a single dataset alone.

The primary aim of this study was to compare the risk-adjusted odds of hospitalisation between bicyclists and motorcyclists involved in crashes. Hospitalisation was used as the study outcome for both bicyclists and motorcyclists to evaluate whether various factors (e.g., demographic characteristics, road and weather conditions, and vehicle characteristics) are associated with hospital admission of casualties sustaining serious or fatal injuries.

## Method

### Data sources

The current research relies on secondary data analysis for the years between 2003 and 2012. The data sources used in the present study were a police-reported crash data set and the National Health Insurance Research Database (NHIRD) from the Health and Welfare Data Science Center, Ministry of Health and Welfare, Taiwan. The police-reported crash data are recorded by the National Police Agency, Taiwan. After every road traffic-related accident of which the police are aware, qualified and experienced police accident investigators complete police-reported crash report forms comprising three files called accident, vehicle and victim, and contributory factor files.

Accident files contain general information regarding the times and dates of crashes; weather, road, and lighting conditions; and road type. Furthermore, vehicle and victim files are used to record information regarding vehicles; riders and victims, such as age, sex, and injury severity; vehicle type; the first point of vehicle impact; and vehicle manoeuvres. There is actually an issue on the validity of injury-severity data from the police-reported crash dataset in Taiwan. Hospitalisation data that are more reliable for analysis of injuries are therefore matched to police accident reports in the current research. The current research concerns with crashes that resulted in injuries to motorcyclists or bicyclists. Crashes that cause property damage only (that are not available in police-reported dataset) are not the focus. Omitting such crashes should not impact our results much as the number of these particular crashes can be comparative few.

The National Health Insurance program was implemented by the Bureau of National Health Insurance (BNHI) in Taiwan on March 1, 1995; it covers medical insurance for 99% of all Taiwan residents. The NHIRD contains national outpatient, emergency, and hospitalisation data, and all hospitals and clinics are required to report to the BNHI on a monthly basis. The BNHI ensures the accuracy of claim files through periodical expert reviews of a random sample of every 50–100 ambulatory and inpatient claims. The information and data from the NHIRD can be considered complete and accurate [33, 34]. The variables present in the NHIRD include inpatient age, sex, admission and discharge dates, care location, hospital level, department of treatment, surgical procedure history, medical expenditure, disease or injury diagnoses (according to the International Classification of Diseases, Ninth Revision, Clinical Modification [ICD-9-CM] N codes), and cause of injury (according to ICD-9-CM E codes). Injury diagnoses are coded using ICD-9-CM N codes 800–999. ICD-9-CM E codes defining motorcycle- or bicycle-related injuries are listed as follows: E800.3, E801.3, E802.3, E803.3, E804.3, E8053, E806.3, E807.3, E810.x-E819.x, E820.6, E821.6, E822.6, E823.6, E824.6, E825.6, E826.1, E826.9, E827.1, E828.1, and E829.1.

Scrambled patient identification codes (IDs) in the NHIRD were used to link patient data externally to the police-reported crash data. Our study was exempted from review by an institutional review board because the patient IDs were encrypted, rendering identification of individual patients or casualties impossible (IRB#:201409033).

There are well-known issues on the completeness of police-reported data. However, in Taiwan, by law all motorised vehicles have “Compulsory Insurance” that provides victims with compensation for injury. Those involved in crashes need to report to police in order to claim compensation for injury. The reviewer is definitely right that under-reporting of crashes can be an issue but we suppose that it would not impact our study significantly. There are also issues on the validity of police-reported data. A typical example includes mobile phone use and BAC level. Data on mobile phone use were not used in the current research so there is no need to concern with it. As for BAC level, in Taiwan by law, drivers/riders must be tested for BAC level if there are casualties in the accident.

**Fig 1:**
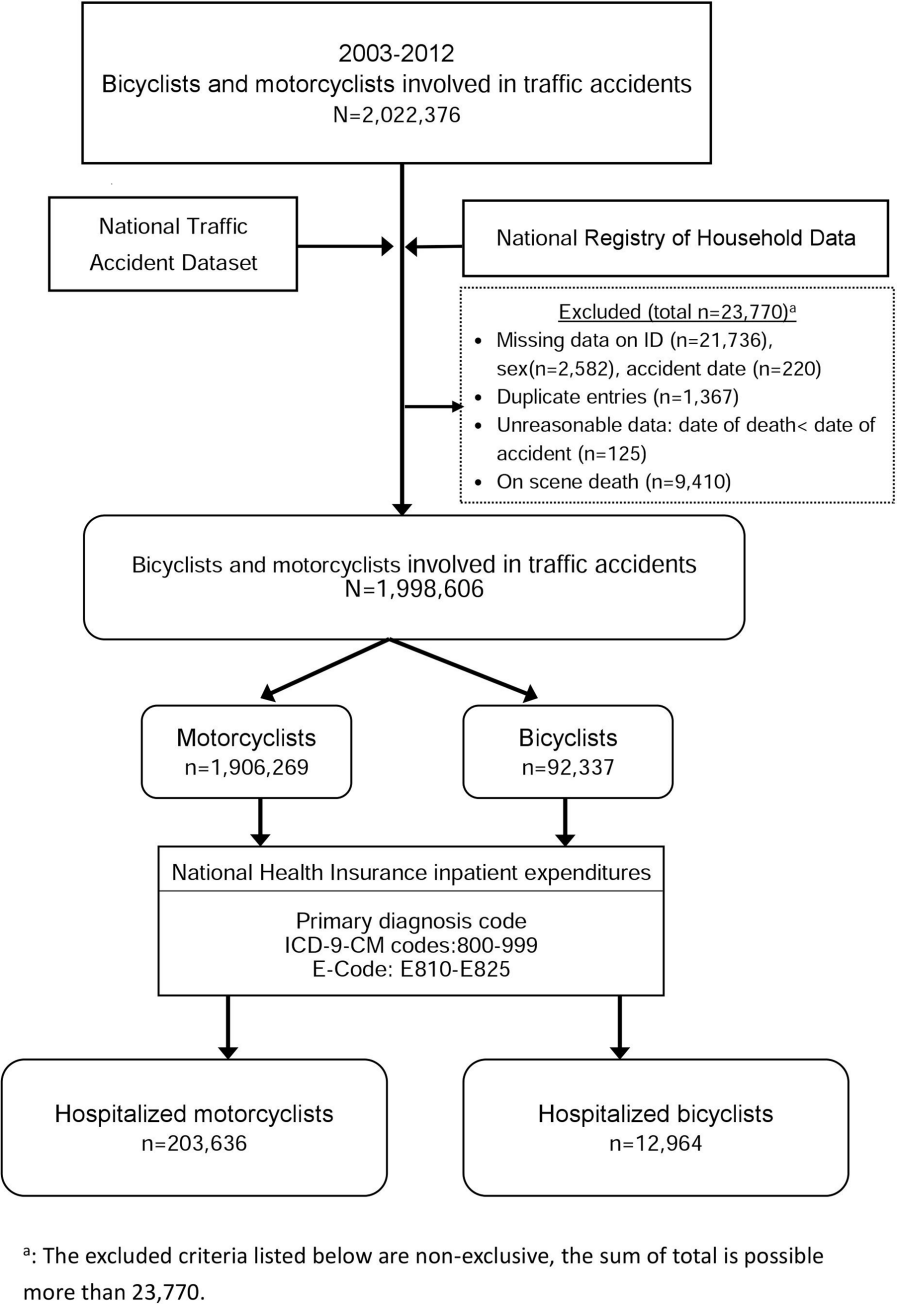
Study flow diagram.

The process of sample selection from the police-reported crash data set and the NHIRD is presented in Fig 1. This study analysed data for the period 2003–2012. A total of 2,022,376 motorcyclist and bicyclist casualties were extracted from police-reported data. Missing data on IDs (n = 21,736), genders (n = 2,582), and accident date (n = 220) were excluded. Duplicate entries (n = 1,367) and unreasonable data (i.e., date of death was earlier that date of crash, n = 125) were removed. Finally, after removing on-scene deaths (n = 9,410), the police-reported data were merged with that NHIRD data (E810-E825), resulting in a total of 1,998,606 bicyclists and motorcyclists (1,906,269 are motorcyclist casualties, and 92,337 are cyclist casualties). Among 1,906,269 motorcyclist casualties, 203,636 were hospitalised. Among 92,337 bicyclist casualties, 12,964 were hospitalised.

It merits mention here that the primary focus of the study was to investigate the influential factors on non-fatal injury, hospitalisation, which is a significant burden on medical treatments. Those who are hospitalised are considered to sustain more severe injuries than those not hospitalised. Using hospitalisation as an injury outcome among motorcyclists and cyclists provides another important injury-severity indicator as opposed to reliance on typical discrete injury-severity levels.

### Definition of variables

The examined demographic data for the patients were sex (male and female), age (four groups: <18, 18–40, 41–64, ≥65 years), marital status (married and single, divorced, or other), blood alcohol concentration (BAC; <0.03% and ≥0.03%), and helmet use (yes and no). Those aged less than 18 are generally identified as teenagers that cannot ride motorcycles legally. Those aged 65 or above are generally identified as the elderly. As for age intervals 18–40 and 41–64, we simply classify the remaining ages into two even age intervals. In Taiwan by law, BAC > 0.03 is considered to be drink-driving and a fine ticket is therefore issued. Drivers/riders who have BAC > 0.03 and get involved in crashes are charged with offense against public safety.

Vehicle attributes were engine size (≤50 and ≥51 cc) and the type of the other vehicle in the crash (bicycle, motorcycle, car or taxi, bus or coach, and heavy goods vehicle). In Taiwan, there are four different engine sizes: engine size up to 50cc, 51cc-250cc, 251cc-550cc, and 551cc or above. In our linked data, there are limited cases for those riding 251cc-550cc and 551cc or above. The limited sample size was primarily because heavy motorcycles (251cc or above) were imported after years 2008 –the population has been much smaller than other small motorcycles (i.e., < = 250cc). The engine size variable therefore has two levels: > = 51cc VS < = 50cc.

Road and environmental factors comprised several variables—location (highly urbanized area, moderately urbanized area, boomtown, general township, and rural area), path type (straight road, curved road, and crossroads or roundabout road), lighting (daylight, dusk, and dawn), road type (provincial highway, county road, and other), road surface (dry and wet or slippery), road defect (yes and no), barrier (yes and no), traffic signal (yes and no), median strip (yes and no), and traffic island (yes and no). The “Location” variable is based on several parameters: population density, population ratio of people with educational degree college or above, population ratio of the elderly, population ratio of agricultural workers, and the number of physicians per 100,000 people. The township classification has been widely adopted in Taiwan (see the study of Liu et al [35]). Provincial roadway is for speed limit of 70km/h, county roadway is for 60km/h, and township roadway is for 50km/h. Uneven roadways or roads with potholes are classified as road defects.

Crash characteristics were the crash type (multiple and single vehicle crashes), object type (unfixed and fixed objects), fixed objects (building or barrier; traffic island, tree, or pole; and other), unfixed objects (animal or pedestrian and skidding). Multi-vehicle and single-vehicle cases involving bicyclists and motorcyclists were included in the study.

### Statistical analysis

Hospitalisation trend of two-wheeled vehicle riders involved in traffic crashes is compared and the difference in hospitalisation percentages is tested with the Mann-Kendall trend test. Distribution of the hospitalised and non-hospitalised casualties by a set of variables (e.g., human attributes, environmental factors and vehicle characteristics) is reported. Chi-square tests are conducted for comparing between hospitalised vs non-hospitalised patients. VIF (variance inflation factor) was conducted to assess multicollinearity among the variables.

This study investigated whether the motorcyclists and bicyclists were hospitalised. Because the dependent variable was binary (hospitalisation vs. no hospitalisation), logistic regression models were used to examine the determinants of hospitalisation. A pooled logistic regression model was estimated using the casualty type (bicyclist vs. motorcyclist) as a variable; in addition, two other models were used in separately examining the determinants of hospitalisation for bicyclists and motorcyclists to reveal the contributory factors varying between bicyclist and motorcyclist casualties. In estimating the models, the variables that have significance level (p<0.2) in the univariate models were then incorporated into the multivariate models. Only confounding variables were included in the models.

## Results

**Fig 2:**
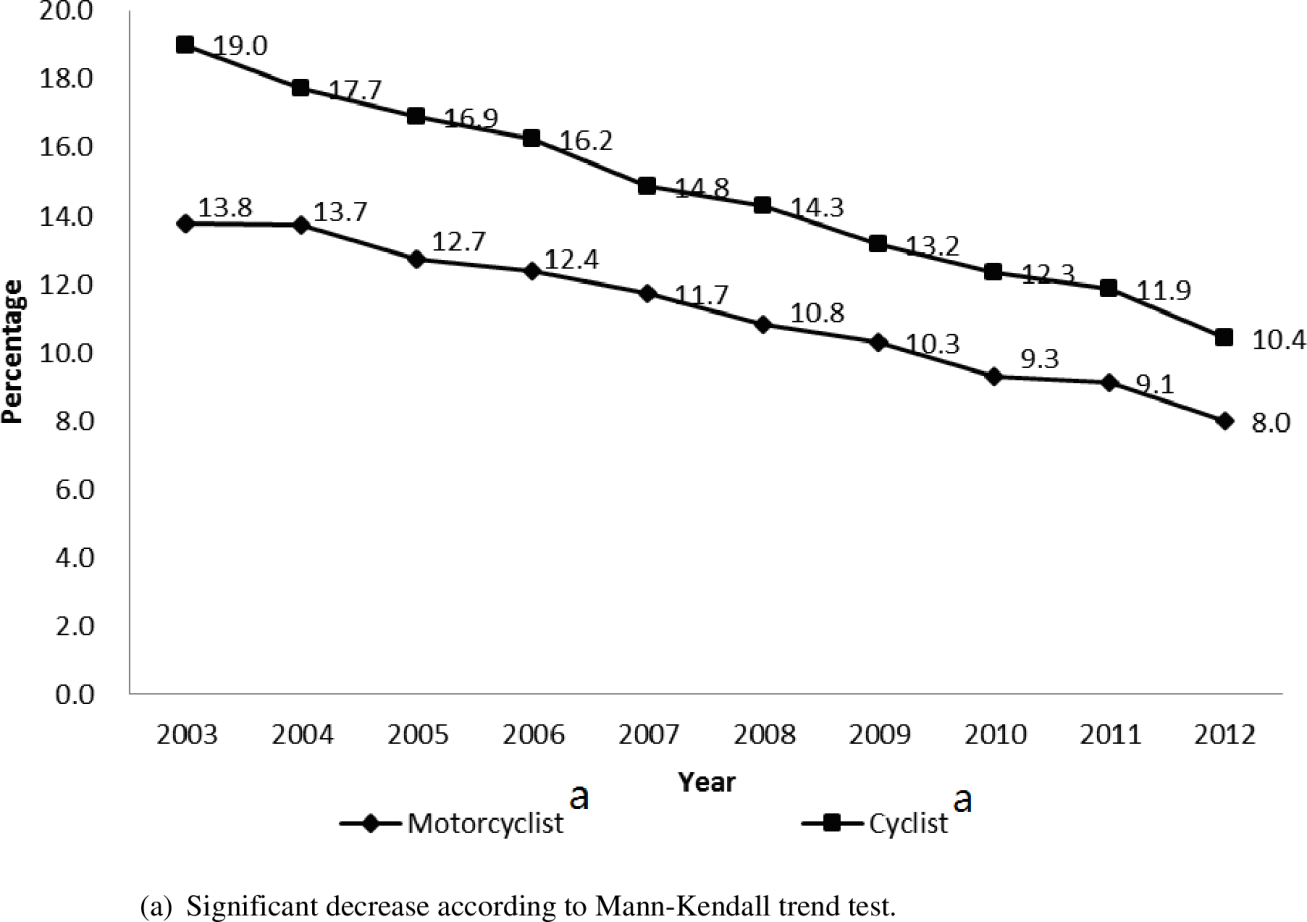
Hospitalisation trend of two-wheeled vehicle riders involved in traffic crashes. (a) Significant decrease according to Mann-Kendall trend test.

Fig 2 presents the trend of hospitalisation among motorcyclists and bicyclists: the trend significantly decreased from 13.8% and 19% in 2003 to 8% and 10.4% in 2012, respectively, according to the Mann-Kendall trend test. In general, there is an overall improvement in safety among bicyclists and motorcyclists over the sample period. Specifically, the hospitalisation percentage was higher among bicyclists than among motorcyclists.

Tables 1–3 provide a summary of the inpatient attributes, environment factors, and vehicle characteristics during 2003–2012. Notably, Table 1 illustrates that on average, bicyclists were more likely to be hospitalised than motorcyclists were (14.0% vs. 10.7%). Furthermore, compared with the other age groups, those aged ≥65 years had the greatest hospitalisation percentage in both motorist groups, and motorcyclists with a BAC < 0.03% exhibited a higher hospitalisation percentage than motorcyclists with a BAC ≥ 0.03% did. In addition, hospitalisation percentages were higher among motorcyclists and bicyclists not wearing helmets than among those wearing them (14.3% and 13.3% vs. 11.8% and 10.4%, respectively). Moreover, the hospitalisation percentage of unlicensed motorcyclists (16.5%) was higher than that of licensed motorcyclists.

**Table 1:** Demographic characteristics of inpatients involved in two-wheeled vehicle crashes.

	Motorcyclists				Bicyclists			
	N^a^	n^b^	%	*P*	N^a^	n^b^	%	*P*
Total	1,906,269	203,636	10.7		92,337	12,964	14.0	
Sex								
Male	1,135,582	116,597	10.3	<0.001	55,534	7,178	12.9	<0.001
Female	770,687	87,039	11.3		36,803	5,786	15.7	
Age group (years)								
<18	60,417	8,001	13.2	<0.001	27,879	2,642	9.5	<0.001
18–40	1,215,460	95,175	7.8		12,792	937	7.3	
41–64	497,477	73,133	14.7		25,607	3,702	14.5	
≥65	132,835	27,321	20.6		26,038	5,682	21.8	
Marital status								
Married	669,741	93,445	14.0	<0.001	35,072	6,320	18.0	<0.001
Single/divorced/other	1,200,989	108,454	9.0		52,333	6,371	12.2	
Location								
Highly urbanized area	419,415	27,303	6.5	<0.001	16,684	1,536	9.2	<0.001
Moderately urbanized area	669,647	61,068	9.1		28,165	3,515	12.5	
Boomtown	432,790	49,822	11.5		20,717	2,961	14.3	
General township	266,896	42,384	15.9		17,768	3,166	17.8	
Rural area	116,922	22,962	19.6		8,978	1,782	19.8	
Motorcycle engine capacity (cc)								
≥51	1,585,249	162,107	10.2	<0.001	NA	NA	NA	NA
≤50	321,020	41,529	12.9		NA	NA	NA	
Intoxicated driving								
No (BAC ≤ 0.03%)	1,808,618	185,128	10.2	<0.001	90,430	12,735	14.1	0.010
Yes (BAC > 0.03%)	97,643	18,508	19.0		1,907	229	12.0	
Helmet use								
Yes	1,726,774	179,745	10.4	<0.001	7,821	919	11.8	<0.001
No	179,490	23,891	13.3		84,516	12,045	14.3	
Licensed								
Yes	1,629,582	158,698	9.7	<0.001	NA	NA	NA	NA
No	239,148	39,416	16.5		NA	NA	NA	

BAC, blood alcohol concentration; NA, not available. There are some missing data for variables such as age, marital status, location, BAC level etc. Therefore, the numbers of cases across some variables are not consistent.

^a^ total number of traffic crashes: 1,998,606 motorcyclists and cyclists

^b^ number of hospitalisations due to traffic crashes: 216,600

**Table 2:** Environmental characteristics of two-wheeled vehicle crashes.

	Motorcyclists				Bicyclists			
	N^a^	n^b^	%	*P*	N^a^	n^b^	%	*P*
Path type								
Straight road	673,458	75,811	11.3	<0.001	40,692	5,820	14.3	0.017
Curved road	69,858	10,002	14.3		3,903	582	14.9	
Crossroads/roundabout	1,162,953	117,838	10.1		47,742	6,563	13.7	
Lighting								
Daylight	1,865,523	198,096	10.6	<0.001	88,827	12,414	14.0	0.005
Dusk/dawn	40,741	5,540	13.6		3,510	550	15.7	
Road type								
Provincial highway	98,538	15,783	16.0	<0.001	5,415	957	17.7	<0.001
County road	133,543	20,014	15.0		7,188	1,348	18.8	
Other (township/private road)	1,674,026	167,839	10.0		79,726	10,659	13.4	
Road surface								
Dry	1,690,159	183,158	10.8	<0.001	83,161	11,762	14.1	<0.001
Wet/slippery	216,110	20,476	9.5		9,176	1,202	13.1	
Defective road								
No	1,887,479	201,036	10.7	<0.001	91,705	12,858	14.0	0.047
Yes	18,790	2,600	13.8		632	106	16.8	
Barrier								
No	1,846,594	196,736	10.7	<0.001	89,794	12,627	14.1	0.246
Yes	59,672	6,900	11.6		2,543	337	13.3	
Traffic signal								
Yes	717,629	70,100	9.8	<0.001	26,543	3,598	13.6	0.007
No	1,188,634	133,536	11.2		65,794	9,366	14.2	
Median strip								
Yes	1,062,575	113,146	10.6	0.087	56,516	7,957	14.1	0.666
No	843,694	90,490	10.7		35,821	5,007	14.0	
Traffic island								
Yes	515,120	57,989	11.3	<0.001	26,938	3,938	14.6	0.001
No	1,391,149	145,647	10.5		65,399	9,026	13.8	

BAC, blood alcohol concentration; NA, not available. There are some missing data for variables such as age, marital status, location, BAC level etc. Therefore, the numbers of cases across some variables are not consistent.

^a^ total number of traffic crashes: 1,998,606 motorcyclists and cyclists

^b^ number of hospitalisations due to traffic crashes: 216,600

**Table 3:** Crash characteristics of inpatients involved in two-wheeled vehicle crashes.

	Motorcyclists				Cyclists			
	N^a^	n^b^	%	*P*	N^a^	n^b^	%	*P*
Crash type								
Multiple vehicle	1,704,253	173,963	10.2	<0.001	88,489	12,393	14.0	0.123
Single vehicle	201,899	29,673	14.7		3,835	571	14.9	
Object type								
Unfixed objects	158,977	19,903	12.5	<0.001	3,142	454	14.4	0.103
Fixed objects	42,922	9,770	22.8		693	117	16.9	
Fixed objects								
Building/barrier	11,402	2,785	24.4	<0.001	102	29	28.4	<0.001
Traffic island/tree/pole	16,325	4,185	25.6		62	16	25.8	
Other	15,195	2,800	18.4		529	72	13.6	
Unfixed objects								
Animal/pedestrian	72,773	4,197	5.8	<0.001	789	36	4.6	<0.001
Skidding	89,346	15,706	17.6		2,353	418	17.8	

BAC, blood alcohol concentration; NA, not available. There are some missing data for variables such as age, marital status, location, BAC level etc. Therefore, the numbers of cases across some variables are not consistent.

^a^ total number of traffic crashes: 1,998,606 motorcyclists and cyclists

^b^ number of hospitalisations due to traffic crashes: 216,600

Regarding vehicle and road characteristics (Tables 2 and 3), motorcyclists and bicyclists exhibited higher hospitalisation percentages when the crashes occurred on curved roadways (14.3% and 14.9%, respectively), in darkness (13.6% and 15.7%, respectively), on dry road surfaces (10.8% and 14.1%, respectively), on defective roadways (13.8% and 16.8%, respectively), and on roads without signals (11.2% and 14.2%, respectively). For both motorist groups, the percentages of hospitalisation resulting from single-vehicle crashes where the vehicle did not collide with another road user were high (14.7% and 14.9%); in addition, the percentages of hospitalisation resulting from single-vehicle crashes where the vehicle collided with fixed objects were high for both motorcyclists and bicyclists (22.8% and 16.9%, respectively).

Table 4 lists crude odds ratios and adjusted odds ratios (AORs) for hospitalisation of motorcyclists and bicyclists determined using logistic regression models. Three models were estimated: one pooled model considering the vehicle type as a risk factor, and two separate models for the determinants of hospitalisation for motorcyclists and bicyclists. Symptoms of multicollinearity (e.g., widely changing coefficients when an additional variable is included or removed and unreasonable coefficient magnitudes) were not observed in the pooled model, and the coefficients of the estimated models had meaningful signs and magnitudes. The variance inflation function (<3) indicated that there is no concern regarding collinearity in the models.

**Table 4:** Crude odds ratios (ORs) and adjusted ORs (AORs) of hospitalisation for motorcyclists and cyclists.

	Two-wheeled vehicle riders				Motorcyclists				Bicyclists			
	Crude OR	95% CI	AOR	95% CI	Crude OR	95% CI	AOR	95% CI	Crude OR	95% CI	AOR	95% CI
Vehicle type												
Motorcycle	1.00 (ref.)		1.00 (ref.)		---		---		---		---	
Bicycle	1.74	1.70–1.77	1.11	1.08–1.14								
Sex												
Male	1.00 (ref.)		1.00 (ref.)		1.00 (ref.)		1.00 (ref.)		1.00 (ref.)		1.00 (ref.)	
Female	1.50	1.48–1.51	1.64	1.62–1.66	1.49	1.48–1.51	1.59	1.58–1.61	1.60	1.53–1.67	1.50	1.43–1.57
Age (years)												
<18	1.11	1.09–1.14	1.53	1.49–1.57	1.17	1.14–1.20	1.37	1.32–1.41	0.50	0.48–0.53	1.41	1.29–1.55
18–40	1.00 (ref.)		1.00 (ref.)		1.00 (ref.)		1.00 (ref.)		1.00 (ref.)		1.00 (ref.)	
41–64	1.68	1.67–1.70	1.79	1.76–1.81	1.73	1.71–1.75	1.74	1.71–1.76	0.99	0.95–1.04	2.19	2.00–2.40
≥65	3.36	3.31–3.41	3.86	3.79–3.93	3.22	3.17–3.28	3.55	3.48–3.62	3.45	3.29–3.62	4.83	4.41–5.30
Marital status												
Married	1.00 (ref.)		1.00 (ref.)		1.00 (ref.)		1.00 (ref.)		1.00 (ref.)		1.00 (ref.)	
Single/divorced/other	0.57	0.56–0.57	0.89	0.88–0.90	0.57	0.56–0.58	0.89	0.88–0.90	0.51	0.49–0.53	0.82	0.77–0.86
Location												
Highly urbanized area	1.00 (ref.)		1.00 (ref.)		1.00 (ref.)		1.00 (ref.)		1.00 (ref.)		1.00 (ref.)	
Moderately urbanized area	0.74	0.73–0.75	1.46	1.44–1.48	0.74	0.73–0.75	1.46	1.44–1.49	0.80	0.76–0.84	1.60	1.48–1.72
Boomtown	1.17	1.15–1.18	1.96	1.92–1.99	1.17	1.16–1.19	1.96	1.93–2.00	1.06	1.01–1.11	2.11	1.95–2.28
General township	2.03	2.01–2.06	2.72	2.67–2.77	2.05	2.02–2.07	2.74	2.69–2.80	1.60	1.52–1.68	2.63	2.42–2.85
Rural area	2.55	2.50–2.59	3.07	3.00–3.14	2.58	2.54–2.63	3.09	3.02–3.16	1.76	1.64–1.88	2.73	2.48–3.01
Motorcycle engine size (cc)												
≥51	---		---		1.00 (ref.)		1.00 (ref.)		---		---	
≤50					1.41	1.40–1.43	1.11	1.09–1.13				
Intoxicated driving												
No (BAC ≤ 0.03%)	1.00 (ref.)		1.00 (ref.)		1.00 (ref.)		1.00 (ref.)		1.00 (ref.)		1.00 (ref.)	
Yes (BAC > 0.03%)	1.97	1.94–2.01	1.95	1.91–1.99	2.05	2.02–2.09	1.86	1.82–1.90	0.60	0.52–0.70	0.70	0.59–0.83
Helmet use												
Yes	1.00 (ref.)		1.00 (ref.)		1.00 (ref.)		1.00 (ref.)		1.00 (ref.)		1.00 (ref.)	
No	1.44	1.42–1.45	1.11	1.10–1.13	1.30	1.28–1.32	1.14	1.12–1.16	1.21	1.12–1.31	1.03	0.94–1.12
Licensed												
Yes	---		---		1.00 (ref.)		1.00 (ref.)		---		---	
No					1.71	1.69–1.73	1.22	1.20–1.24				
Path type												
Straight road	1.00 (ref.)		1.00 (ref.)		1.00 (ref.)		1.00 (ref.)		1.00 (ref.)		1.00 (ref.)	
Curved road	1.55	1.51–1.58	1.41	1.38–1.45	1.57	1.54–1.61	1.43	1.39–1.46	1.08	0.98–1.20	1.25	1.11–1.40
Crossroads/roundabout	0.87	0.86–0.88	1.01	1.00–1.03	0.87	0.86–0.88	1.01	0.99–1.02	1.03	0.99–1.08	1.12	1.05–1.19
Lighting												
Daylight	1.00 (ref.)		1.00 (ref.)		1.00 (ref.)		1.00 (ref.)		1.00 (ref.)		1.00 (ref.)	
Dusk/dawn	1.44	1.40–1.49	1.04	1.01–1.08	1.44	1.40–1.49	1.04	1.01–1.08	1.11	1.00–1.23	1.05	0.93–1.18
Road type												
Provincial highway	1.00 (ref.)	1.00 (ref.)	1.00 (ref.)		1.00 (ref.)	1.00 (ref.)	1.00 (ref.)		1.00 (ref.)	1.00 (ref.)	1.00 (ref.)	1.00 (ref.)
County road	1.71	1.68–1.74	0.95	0.93–0.98	1.71	1.68–1.74	0.95	0.92–0.97	1.65	1.53–1.78	1.09	0.96–1.23
Other (Township/private road)	0.53	0.52–0.54	0.75	0.74–0.77	0.53	0.52–0.53	0.75	0.73–0.76	0.62	0.58–0.65	0.84	0.76–0.93
Road surface												
Dry	1.00 (ref.)		1.00 (ref.)		1.00 (ref.)		1.00 (ref.)		1.00 (ref.)		1.00 (ref.)	
Wet/slippery	0.88	0.86–0.89	0.92	0.90–0.93	0.88	0.86–0.89	0.92	0.90–0.93	0.93	0.86–1.00	0.98	0.91–1.06
Defective road												
No	1.00 (ref.)	1.00 (ref.)	1.00 (ref.)		1.00 (ref.)	1.00 (ref.)	1.00 (ref.)		1.00 (ref.)	1.00 (ref.)	1.00 (ref.)	1.00 (ref.)
Yes	1.59	1.52–1.66	1.33	1.26–1.40	1.62	1.54–1.69	1.34	1.27–1.41	1.22	0.95–1.55	1.35	1.03–1.76
Barrier												
No	1.00 (ref.)		1.00 (ref.)		1.00 (ref.)		1.00 (ref.)		1.00 (ref.)		1.00 (ref.)	
Yes	1.09	1.06–1.12	0.99	0.96–1.02	1.11	1.08–1.14	1.00	0.97–1.03	0.88	0.77–1.00	0.89	0.77–1.03
Traffic Signal												
Yes	1.00 (ref.)		1.00 (ref.)		1.00 (ref.)		1.00 (ref.)		1.00 (ref.)		1.00 (ref.)	
No	1.17	1.16–1.18	1.01	0.99–1.02	1.17	1.16–1.18	1.01	0.99–1.02	1.01	0.96–1.06	0.96	0.90–1.02
Median strip												
Yes	1.00 (ref.)		1.00 (ref.)		1.00 (ref.)		1.00 (ref.)		1.00 (ref.)		1.00 (ref.)	
No	0.98	0.97–0.99	1.13	1.12–1.15	0.99	0.98–1.00	1.13	1.11–1.14	1.00	0.96–1.04	1.07	1.01–1.13
Traffic island												
Yes	1.00 (ref.)		1.00 (ref.)		1.00 (ref.)		1.00 (ref.)		1.00 (ref.)		1.00 (ref.)	
No	0.93	0.92–0.94	0.84	0.83–0.85	0.93	0.92–0.94	0.85	0.83–0.86	1.00	0.95–1.04	0.93	0.87–0.98
Crash type												
Multiple vehicle	1.00 (ref.)		1.00 (ref.)		1.00 (ref.)		1.00 (ref.)		1.00 (ref.)		1.00 (ref.)	
Single vehicle	1.47	1.45–1.49	1.29	1.27–1.31	1.51	1.49–1.54	1.29	1.27–1.31	0.87	0.79–0.97	0.97	0.87–1.09

BAC, blood alcohol concentration; CI, confidence interval.

One significant result in the pooled model was that the odds of hospitalisation were greater in bicyclists when compared to motorcyclists (AOR = 1.11). Other factors significantly associated with hospitalisation were the female sex, an age of 65 years or older, rural areas, a BAC ≥ 0.03%, helmet non-use, curved roadways, darkness, defective roadways, no median strip, and single-vehicle crashes.

In the motorcyclist and bicyclist models for estimating factors contributing to hospitalisation, the estimated crude ORs and AORs were identical to those of the pooled model, except for the BAC. BAC > = 0.03% appeared to be a risk factor for hospitalisation for only motorcyclists (AOR = 1.86): drunken motorcyclists had an 86% in the probability of hospitalisation than sober ones. The alcohol effect appears to be a protective factor for bicyclists.

Female (AORs = 1.59 and 1.50) and elderly (AORs = 3.55 and 4.83) two-wheeled vehicle riders had higher odds of hospitalisation. Crashes occurring in rural areas were associated with higher odds of hospitalisation in motorcyclists and bicyclists (AORs = 3.09 and 2.73, respectively). Riding without a helmet was a significant risk factor for motorcyclists (AOR = 1.14) but the effect appears insignificant for cyclists. It shows here that unhelmeted motorcyclists had a 29% increase in the probability of hospitalisation than helmeted ones. Riding without a legal license increased the probability of hospitalisation by 22% (AOR = 1.22). Curved roadways were associated with higher odds of hospitalisation in motorcyclists and bicyclists (AORs = 1.43 and 1.25, respectively). Motorcyclists and bicyclists involved in crashes on defective roadways had a 34% and 35% increase in the probabilities of hospitalisation (AORs = 1.34 and 1.35) than those involved in crashes on non-defective roadways, respectively. Motorcyclists involved in single-vehicle crashes had a 29% increase in the probability of hospitalisation than those involved in multivehicle crashes were (AOR = 1.29). By contrast, single-vehicle crashes were protective factors against hospitalisation for bicyclists (AOR = 0.97); however, the effect was non-significant.

## Discussion and conclusions

By using the police-reported crash data set and National Health Insurance Research Database, this study successfully identified several factors associated with the increased odds of hospitalisation in motorcyclists and bicyclists in Taiwan. The pooled model, in which both groups (motorcyclists vs. bicyclists) were considered the independent variable, revealed that bicyclists have an 11% increased probability of being hospitalised relative to that of motorcyclists. This finding is particularly noteworthy because the recent emphasis on cycling as an alternative transportation mode to automobile transportation has necessitated research on bicycle safety regarding sharing roadways with motorized vehicles. A potential reason for the aforementioned statistics is that in Taiwan, helmet use has been legally mandatory for motorcyclists since 1997 (although the enforcement remains more lenient for bicyclists), leading to significant decreases in the numbers of fatalities and head injuries sustained by motorcyclists [36];this reason should be further examined using additional exposure data.

The estimation results of the two separate logistic models of motorcyclists and bicyclists demonstrated a similar pattern of the factors contributing to hospitalisation among the two groups. However, although the BAC >0.03% was a determinant of hospitalisation for motorcyclists, it was a protective factor for bicyclists. In motorcyclists, the aforementioned effect of the BAC is justified because the reaction ability of intoxicated motorcyclists may be impaired, potentially aggravating their injury outcomes and consequently increasing their hospitalisation odds [37]. Our data suggest an increased hospitalisation likelihood among drunken motorcyclists (19% versus 10%). The protective effect of BAC on bicyclists can perhaps be explained by speed, which has a critical role—intoxicated bicyclists may not ride as fast as sober bicyclists do, reducing their hospitalisation percentages. Nevertheless, our results do not encourage riding bicycles while intoxicated, because intoxicated bicyclists may still cause road safety problems.

The current study demonstrated that elderly and female motorcyclists and bicyclists exhibit greater hospitalisation odds, corroborating the results of previous studies on motorcyclist and bicyclist injury severity [17, 38]. Males are generally physically stronger than females, and elderly road users typically have more complications than younger road users do; thus, the percentages of hospitalisation after an accident are higher among female and elderly people [39]. Education efforts should be directed toward elderly road users—they should be informed regarding their increased injury severity and higher hospitalisation odds after a crash.

Although no data regarding riding speeds are available, higher crash velocities, common in single-vehicle crashes, may lead to higher hospitalisation percentages. Speed management schemes targeting all motorized vehicles, particularly motorcycles, may be effective countermeasures for reducing hospitalisation percentages. Motorcyclists and bicyclists require physical balance when riding; thus, they are likely to lose control on defective roadways. Therefore, our finding that defective roadways are associated with higher hospitalisation percentages suggests the need for improving roadway surfaces and providing initial warnings informing motorcyclists and bicyclists of dangers ahead.

The present study also indicated that crashes occurring in rural areas are associated with higher hospitalisation odds in motorcyclists and bicyclists. Our results are justified by the higher kinetic energy of vehicles and greater impact at higher speeds in rural areas [17, 40]. Other possible reasons for this finding include the additional time required for emergency vehicle response and lower availability of medical resources in rural areas [41]. A specific comparison of AORs between motorcyclists and bicyclists indicated relative higher hospitalisation percentages among motorcyclists (AOR = 3.09), implying that the effect of rural areas(i.e., higher speed limit and crash velocities and lower availability of medical treatment)on motorcyclists is more pronounced than that on bicyclists.

Our results show that moped users (< = 50cc) had higher odds of hospitalisation than users of large motorcycles (>50cc). Rider ages (young/elderly riders are the main group of scooter users) can be the contributing factor to the increased hospitalisation odds. That is, young riders tend to have risk-taking behaviours and they are more likely to wear tropical helmets that are cheaper and have poorer protection. Elderly riders are more vulnerable to injuries and therefore are more likely to be hospitalised once they are involved in an accident. A better understanding of helmet wearing and types of helmet worn by users of different ages in a motorcycle dominated country like in Taiwan is a fruitful area for future research. It also merits mention here that by law, the requirement for legally riding smaller motorcycles (up to 50cc) was to pass the knowledge tests (paper exam only) and no road test was required, which was possibly the contributory factor to the higher hospitalisation odds. The more stringent license law has been implemented in year 2017, requiring that a road test is compulsory.

Here, helmet non-use was also a risk factor for motorcyclists (AOR = 1.14); however, the results were non-significant for bicyclists. Studies [18, 42] have reported an association between riding without a helmet and head injuries or fatalities; our findings regarding the increased hospitalisation percentage emphasize the importance of helmet use.

Our finding that unlicensed motorcyclists are more prone to hospitalisation than are licensed motorcyclists can be explained by two factors. First, inexperienced riders, such as teenage motorcyclists, are more likely to ride unlicensed [43] and engage in risk-taking behaviours, thus increasing their odds of hospitalisation after a crash. Second, obtaining a license for using motorcycles with an engine size of 400cc or greater is difficult; unlicensed riding, along with greater engine power output from large motorcycles, may predispose the riders to higher hospitalisation odds. The aforementioned assumptions must be confirmed by examining the interaction effects among engine size, rider age, and license status.

Regarding the effect of curved roadways, the results indicating that both motorcyclists and bicyclists are more prone to hospitalisation cannot be explained clearly. Crashes in which the vehicle is run off the road are likely to occur on curved roadways and tend to cause substantial injury. In addition, decreased visibility and limited sight distance on curved roadways may impair motorcyclists’ control, thereby increasing their percentage of hospitalisation after a crash.

The interpretation of our results should proceed with caution, because a certain amount of single-vehicle crashes can be off-road crashes and are seldom reported to police. As Meuleners et al. reported, the underreporting of single-vehicle crashes was more prevalent in rural areas, and the adjusted risk of hospitalisation increased five times when compared to urban locations [44]. In our study, an increased likelihood of hospitalisation was similarly found for motorcyclists (AOR = 1.29) in single-vehicle crashes but not for cyclists. The collection of injury/crash data on these single-vehicle crashes, for instance, off-road crashes, that are not available from police-reported dataset could provide important additional insights, and we view this avenue as the most fruitful for future research.

The main strength of this population-based study pertains to the use of two data sets, the police-reported crash data set and NHIRD, which provided reliable data. Thus, the findings of this study are more reliable compared with those of studies relying on police-reported crash data sets alone. However, this study has some limitations. It should be noted here that our data do not include all crashes. Instead, our data only include those that are reported to police in order to claim compensation for injury. Therefore, the current research includes a cohort of patients that have injuries significant enough to warrant compensation. Another major limitation pertains to the data and variables unavailable in the two data sets; the unavailable data and variables include the speeds of motorcycles and other vehicles, traffic volume, geometric characteristics, and electronic device (e.g., phone and MP3 players) use, which may aggravate injury outcomes and increase hospitalisation percentages.
